# Antibacterial Character of Cationic Polymers Attached to Carbon-Based Nanomaterials

**DOI:** 10.3390/nano10061218

**Published:** 2020-06-22

**Authors:** Daniela Plachá, Alexandra Muñoz-Bonilla, Kateřina Škrlová, Coro Echeverria, Alberto Chiloeches, Martin Petr, Khalid Lafdi, Marta Fernández-García

**Affiliations:** 1 Nanotechnology Centre, VŠB–Technical University of Ostrava, 17. listopadu 2172/15, 708 00 Ostrava-Poruba, Czech Republic; katerina.skrlova@vsb.cz; 2 Centre ENET, VŠB–Technical University of Ostrava, 17. listopadu 2172/15, 708 00 Ostrava-Poruba, Czech Republic; 3 Instituto de Ciencia y Tecnología de Polímeros (ICTP-CSIC), C/Juan de la Cierva 3, 28006 Madrid, Spain; sbonilla@ictp.csic.es (A.M.-B.); cecheverria@ictp.csic.es (C.E.); achiloeches@ictp.csic.es (A.C.); 4 Interdisciplinary Platform for Sustainable Plastics towards a Circular Economy-Spanish National Research Council (SusPlast-CSIC), 28006 Madrid, Spain; 5 Regional Centre of Advanced Technologies and Materials, Faculty of Science, Palacký University Olomouc, 17. listopadu 1192/12, 771 46 Olomouc, Czech Republic; martin.petr@upol.cz; 6 Department of Mechanical and Construction Engineering, Northumbria University, Newcastle upon Tyne NE1 8ST, UK; KHALID.LAFDI@NORTHUMBRIA.AC.UK

**Keywords:** graphene oxide, graphene, antimicrobial, polymer, dopamine, cationic group, functionalizations

## Abstract

The preparation of hybrid polymeric systems based on carbon derivatives with a cationic polymer is described. The polymer used is a copolymer of a quaternizable methacrylic monomer with another dopamine-based monomer capable of anchoring to carbon compounds. Graphene oxide and graphene as well as hybrid polymeric systems were widely characterized by infrared, Raman and photoemission X-ray spectroscopies, electron scanning microscopy, zeta potential and thermal degradation. These allowed confirming the attachment of copolymer onto carbonaceous materials. Besides, the antimicrobial activity of hybrid polymeric systems was tested against Gram positive *Staphylococcus aureus* and *Staphylococcus epidermidis* and Gram negative *Escherichia coli* and *Pseudomonas aeruginosa* bacteria. The results showed the antibacterial character of these hybrid systems.

## 1. Introduction

In the last recent years, carbon-based nanomaterials have been explored as novel antimicrobial agents, especially for the preparation of antimicrobial and antibiofouling surfaces [1,2,3,4,5,6,7,8,9]. Some studies have revealed that graphene and its derivatives exhibit excellent antibacterial activity and low mammalian cell toxicity [10], however, the mechanism(s) of this antimicrobial activity still remain controversial [11,12]. Several modes of action have been proposed, e.g., oxidative stress, contact-mediated physical damage and wrapping, although physical properties such as morphology, size, aggregation and surface functionality might affect strongly their antimicrobial activities [13,14,15]. In spite of this, the low solubility of graphene in both organic and aqueous solvents because of its tendency to aggregate, limits its applicability. For this reason, hydrophilic graphene derivatives have been prevailingly used and tested as antimicrobial agents. Among them, graphene oxide (GO) is the hydrophilic graphene derivative that has been most investigated due to its hydrophilicity and its capacity to form homogeneous aqueous dispersions [16,17,18]. Related to graphene and with the purpose of improving its dispersibility, composite systems have been explored, for instance, by incorporating polymers to form a stable dispersion [19]. The preparation of carbon-based composites not only improves the stability and dispersibility of the systems. but also can tune their properties to develop materials with enhanced antimicrobial activities. Graphene and its derivative materials have relatively high specific surface area with abundant surface functionalities in case of graphene oxide, which have the potential for the preparation of multiple composite antibacterial materials in combination with other antimicrobial agents [20,21]. It has been proved the synergistic or additive effect of graphene derivatives with silver composite materials, leading an enhanced activity at low concentration [22,23,24,25]. Similarly, hybrid systems with ZnO and TiO_2_ nanoparticles have demonstrated excellent antibacterial activity [26,27,28]. Polymer-based composites have received special attention due to their versatility and capacity to control their properties. In particular, cationic antimicrobial polymers such as quaternary ammonium polymers, chitosan or polyethyleneimine polymer (PEI) have been widely used in combination with graphene derivatives [29,30,31,32]. The general accepted antimicrobial mechanism of cationic polymers is associated with adsorption on the negatively charged bacterial wall through electrostatic interactions, which augments membrane permeability and subsequently disrupts the membrane. In addition to the antimicrobial activity, the cationic polymers provide stability, dispersibility in aqueous media and could also reduce toxicity. There are three main approaches to develop graphene-polymer composites: physical mixing, covalent bonding of the polymer to graphenic structure and through non-covalent interactions, such as van der Waals forces, hydrophobic interactions, and π-π stacking.

In the present study, we have incorporated a monomer with catechol side chains in a methacrylic cationic polymer, which exhibit excellent antimicrobial activity with the aim to improve the immobilization on graphene derivatives. The copolymer was composed of *N*-(3,4-dihydroxyphenethyl) methacrylamide (DOMA) and 2-(4-methylthiazol-5-yl)ethyl methacrylate (MTA) quaternized with methyl iodide [33]. The randomly distributed DOMA units have the capacity to strongly adhere on graphene and graphene oxide sheets through π-π stacking interaction that stabilizes the dispersion of the nanocomposite. These adhesive units would also be used to bind different surfaces and create antimicrobial coatings. 

## 2. Materials and Methods

### 2.1. Materials

Graphite flakes for graphene oxide (GO) synthesis were supplied by Sigma Aldrich (Praha, Czech Republic). Concentrated sulphuric acid (H_2_SO_4_, 98%), potassium permanganate (KMnO_4_, ≥99%), hydrogen peroxide (H_2_O_2_, 30%) and hydrochloric acid (HCl, 35–37%) were procured from VWR International (Stříbrná Skalice, Czech Republic). Ultra-pure water (Milli Q, Merck, Praha, Czech Republic) was used for synthesis as well as washing.

Monomers N-(3,4-dihydroxyphenethyl) methacrylamide (DOMA) and 2-(4-methylthiazol-5-yl)ethyl methacrylate (MTA) were synthetized according to previous work and used for consequent preparation of the statistical copolymer P(MTA_x_*-co-*DOMA_y_) with monomer molar ratio MTA/DOMA = 90/10 by free radical polymerization (M_n_ = 24,600 g/mol) [33]. The prepared copolymer was then quaternized with iodomethane (MeI, 99%, supplied by Sigma Aldrich) leading the corresponding cationic copolymer bearing thiazolium groups (named as MD10). This quartenized copolymer was used for functionalization of graphene (GR) and GO.

For microbiological studies: sodium chloride aqueous solution (NaCl, 0.9%, BioXtra, suitable for cell cultures) and phosphate buffered saline (PBS, pH 7.4) were purchased from Sigma-Aldrich (St. Louis, MO, USA). BBL™ Mueller–Hinton broth was supplied by Becton, Dickinson and Company (Madrid, Spain) and was used as a microbial growth medium. The 96 well microplates were obtained from Thermo Scientific (Madrid, Spain). Representative bacterial species, two Gram positive *Staphylococcus aureus* (*S. aureus*, ATCC 29213) and *Staphylococcus epidermidis (S. epidermidis,* ATCC 12228) and two Gram negative *Escherichia coli* (*E. coli*, ATCC 25922) and *Pseudomonas aeruginosa* (*P. aeruginosa*, ATCC 27853) were obtained from Oxoid^TM^ (Madrid, Spain) and chosen as model bacterial strains in the present study.

### 2.2. Synthesis of GO and GR

Graphene oxide (GO) was prepared from graphite according to slightly modified Hummers method [34]. Briefly, graphite flakes (10 g) were dispersed firstly in concentrated sulfuric acid solution (250 mL) in an ice bath, then KMnO_4_ (30 g) was slowly added and the mixture was kept stirring for 3 h. Subsequently, the mixture was diluted with distilled water (2 L) followed by adding 30% hydrogen peroxide (50 mL) drop-by-drop. This pre-oxidized product was further oxidized by repeating the previous procedure: pre-product was well mixed with H_2_SO_4_ (250 mL), then KMnO_4_ (30 g), distilled water (2 L) and 30% hydrogen peroxide (50 mL) were gradually added. Finally, the product was washed repeatedly with diluted HCl (1:10) and consequently with demineralized water until pH value of this solution was neutral. The solid phase was then separated and dried overnight at 40 °C to obtain GO. Exfoliated graphite (GR) was prepared by processes of intercalation and exfoliation of graphite flakes with an average diameter of 500 mm [35]. The intercalated material was subjected to heating in a high temperature reactor at 900 °C. Resulted material was grounded after cooling [35]. 

### 2.3. GO and GR Functionalization

The functionalization of GO was conducted in aqueous solution. At first, GO (approx. 70 mg) was properly dispersed in distilled water (30 mL) in an ultrasonic bath for two hours. After sonication, there were no visible GO particles in the mixture. Simultaneously, MD10 polymer (70 mg) was dissolved in distilled water (30 mL). Then, the aqueous suspension of GO was quantitatively added to the polymer solution to prepare a mixture containing GO/polymer ratio 1:1. The mixture was well magnetically stirred for 2 weeks at room temperature. The sample was denoted as GO_MD10. In the case of GR functionalization, the procedure was exactly the same than with GO but the solvent used was DMF. The sample was denoted as GR_MD10. 

After that, both samples were repeatedly washed with distilled water and centrifuged (5810 R centrifuge, Eppendorf, Madrid, Spain) at 12,000 rpm for 30 min to eliminate all non-attached polymers and the DMF solvent in the case of GR. The non-attached polymers elimination in both samples was checked by analysis of obtained supernatants using UV/VIS spectrometer (NanoDrop™One^C^ microvolume UV-Vis spectrophotometer, Thermo Fisher Scientific). Finally, the solid phases were dried at 50 °C until constant weight. 

### 2.4. Characterization of Hybrid Materials 

A Zetasizer Nano ZS (Malvern Instruments, Malvern, United Kingdom) was used to estimate the charge potential of the prepared materials. Samples were dispersed in distilled water (10 μg/mL) and measured at 25 °C. Each measurement was repeated at least ten times. 

Thermogravimetric analysis (TGA) was conducted using a TGA Q500 analyzer (TA Instruments, New Castle, DE, USA) from room temperature to 900 °C at a heating rate of 10 °C/min under an air atmosphere. The instrument was calibrated in temperature and weight by standard methods.

Fourier transform infrared spectroscopy (FTIR) was conducted on a Spectrum Two FT-IR spectrometer (Perkin Elmer, Waltham, MA, USA) in range of 4000–400 cm^−1^ with resolution of 4 cm^−1^. Approximately 2 mg of each sample and 200 mg of KBr (FT-IR purity, spectroscopically dry, Sigma Aldrich) were weighted and transferred to an agate mortar, grinded together and KBr pellets were prepared using a hydraulic press. 

Raman spectra of prepared samples were recorded with a Renishaw inVia Reflex Raman system (Renishaw plc, Wotton-under-Edge, UK) using a grating spectrometer with an 1800 mm^−1^ Peltier-cooled charge-coupled device (CCD) detector, coupled to a confocal microscope operating at an excitation wavelength of 532 nm. Samples were prepared by drop-casting of diluted samples solutions on freshly cleaved SiO_2_/Si wafers and dried at room temperature.

The XPS measurements were carried out with the PHI 5000 VersaProbe II XPS system (Physical Electronics, Chanhassen, MN, USA) with monochromatic Al-Kα source (15 kV, 50 W) and photon energy of 1486.7 eV. All the spectra were measured in the vacuum of 1.4 × 10^−7^ Pa and at 20.5 °C. The analyzed area on the sample had a spot of 100 µm in diameter. The survey spectra were measured with pass energy of 187.850 eV and step of 0.8 eV, while for the high resolution spectra were used pass energy of 23.500 eV and step of 0.2 eV. Dual beam charge compensation was used for all measurements. The spectra were evaluated with the MultiPak (Ulvac-PHI, Inc., Chanhassen, MN, USA) software. All binding energy (BE) values were referenced to the carbon peak C1s at 284.80 eV.

The morphology of the samples were characterized by field emission scanning electron microscopy (FE-SEM) using a SU 8000 microscope (Hitachi, Hitachi, Japan) at 5 kV in transmitted electron imaging mode. Diluted aqueous solutions of the samples were pipetted onto carbon Type-A 400-mesh TEM copper grid obtained from Tedpella Inc. (Redding, CA, USA), and then were dried with filter paper.

### 2.5. Antimicrobial Activity 

Firstly, the bacteria (Gram-positive *Staphylococcus aureus* (*S. aureus*, ATCC^®^ 29213), staphylococcus coagulase-negative *Staphylococcus epidermidis* (*S. epidermidis*, ATCC^®^ 12221) and Gram-negative *Escherichia coli* (*E. coli*, ATCC^®^ 25922) and *Pseudomonas aeruginosa* (*P. aeruginosa*, ATCC^®^ 27853)), were incubated on Columbia Agar plates with 5% sheep blood for 24 h at 37 °C in a IQ050 incubator (Jouan, Winchester, VA, USA). Subsequently, bacteria suspensions of about 10^8^ colony-forming units (CFU) were prepared by adjusting concentration with saline solution to ca. 0.5-0.6 of the McFarland turbidity standard. Suspensions of ca. 2 × 10^6^ CFU mL^−1^ were finally obtained by further dilution with Mueller–Hinton broth. The dispersion of the samples in Mueller-Hinton broth (1 mg/mL) were prepared in glass vials, sonicated and sterilized by UV radiation for 30 min before experiments. Then, 800 µL of bacterial suspension was added to 200 µL sample dispersion to reach a final concentration of 500 µg/mL. Control experiments with only inoculum were also performed. The vials were incubated with gentle shaking during 24 h and then, 200 µL of each solution (without residue of carbon material) were placed in a 96-well plate. Bacterial growth was reflected by the absorption of optical density (OD) at 550 nm via a microplate reader (VirClia^®^ Chemiluminescence). The measurements were made at least in triplicate and the antibacterial ratio was calculated as follows:

$$Antibacterial\;ratio=\frac{\mathrm{OD}\;\mathrm{of}\;\mathrm{control}-\mathrm{OD}\;\mathrm{of}\;\mathrm{sample}}{\mathrm{OD}\;\mathrm{of}\;\mathrm{control}}\times 100$$

## 3. Results

**Figure 1:**
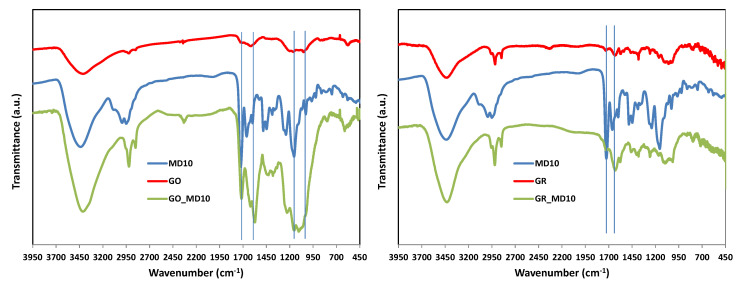
FTIR spectra of hybrid polymeric materials, GO, GR and MD10.

The attachment of polymers to the GO or GR surfaces is evaluated by different techniques. Firstly, FTIR spectra of the carbon-based hybrid materials as well as GO, GR and the copolymer are shown in Figure 1. In the spectra of GO and GR, the presence of oxygenated groups are appreciated. Characteristic vibration and deformation bands at 3410 cm^−1^ (O–H), 1716 (–C=O), 1615 (sp^2^ aromatic C–C), 1164 (sp^2^ aromatic CH in plane) and 1052 cm^−1^ (C–O–C) were observed in the GO pattern. Bands with very low intensity at 3441 (O–H), 1738 (–C=O), 1385 (–C–O), 1065 and 1020 (C–O–C) cm^−1^ are also identified in the GR spectrum. In the case of copolymer, characteristic bands at 3420, 3089, 3001 and 2936 cm^−1^ can be easily assigned to O–H bonds, sp^2^ asymetric vibration of =CH_2,_ –C=N, sp^2^ aromatic CH and sp^3^ asymetric –CH_3_, respectively. The band at ca. 1660 cm^−1^ is associated to –C=O groups of DOMA comonomer, while the band at 1720 cm^−1^ is asignated to –C=O groups of MTA comonomer. The band at 1595 cm^−1^ corresponds to –C=N^+^ of the thiazolium groups of MTA units and the strong band at 1150 cm^−1^ is assignated to C–O groups of MTA units. The attachment of the cationic polymer to obtain the hybrid materials is evident by the presence of carbonyl groups of DOMA and MTA units in each spectrum. 

**Figure 2:**
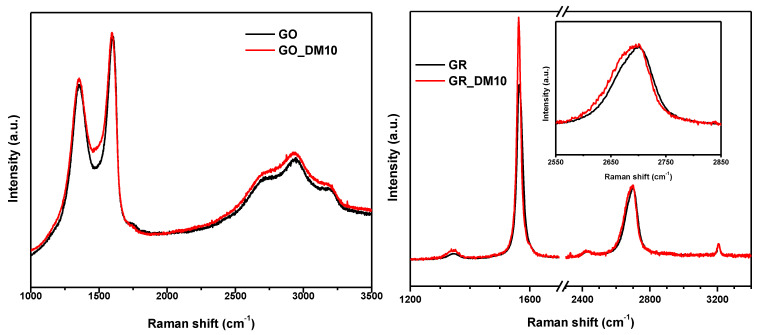
Raman spectra of the hybrid polymeric materials with GO and GR, respectively.

Raman spectra of GO, GR and their hybrid materials are presented in Figure 2. The spectrum of polymer was not possible to measure because of its fluorescence emission. The spectrum of GO shows the typical D (defects inherent in the graphite and the edge effect of graphite, A1g mode) and G (first-order scattering of E2g phonons by sp^2^ carbon atoms) bands at 1350 and 1610 cm^−1^, respectively [36]. There is a broad band in the area around 3000 cm^−1^ that is assigned to 2D, G+D and 2D’ peaks. These bands are characteristics of GO. Meanwhile, the GR spectrum shows that the obtained material is exfoliated graphite. A prominent G band at 1564 cm^−1^ is displayed, the intensity of D band at 1338 cm^−1^ is very low and the intensity ratio between both bands (I_D_/I_G_) is 0.05, which indicates the low presence of defects in the structure [37]. As known, the 2D peak between 2600 and 2800 cm^−1^ in bulk graphite consists of two components 2D_1_ and 2D_2_ [38]. The splitting of this broad Raman band opens up in going from mono- to three-layer graphene and then closes up in going from four layers to highly oriented pyrolytic graphite [39,40]. Intensity ratio of 2D/G peak gives idea about the number of layers; its value is 2 for monolayer, 1 for bilayer, and as this value decreases the number of layers increases. In the case of GR and after deconvolution of 2D peak, this ratio is 0.25, which indicates that GR presents a large number of layers. 

**Figure 3:**
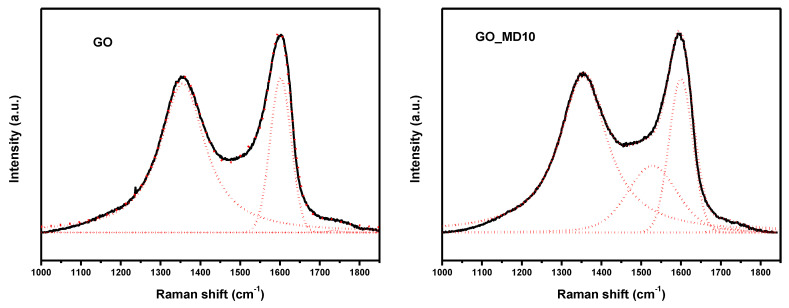
Deconvolution of Raman spectra of GO and GO_MD10 in the range of 1000-1850 cm^−1^.

The Raman spectrum of GO-hybrid polymeric material shows minor changes compare to GO. The spectra are nearly identical; there are no shifts in positions of D and G bands; however, in a deeper inspection by deconvoluting the spectra a new band at 1540 cm^−1^ appears when polymer is attached (see Figure 3). The G and D bands do not change, so the GO structure is maintained, and the new bands could corresponds to –C–O–C– bond from the ring of DOMA and the graphitic structure of GO. 

There are also some substantial changes in GR derivative. There are changes on the intensity of D, G and 2D bands; the ratio of I_D_/I_G_ is a slightly higher 0.08 and also the shape of 2D band has changed (see the insert). These features can correspond to the intercalation of polymer chains between graphitic layers and formation of graphene stacks with less number of layers. 

**Figure 4:**
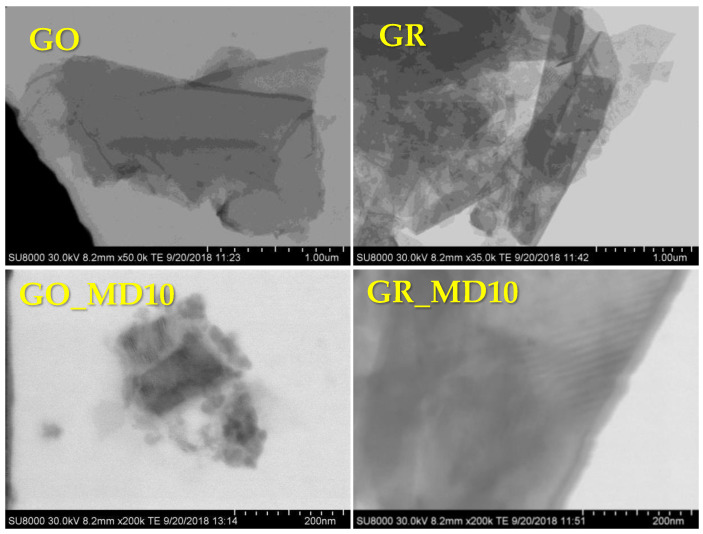
FESEM images of GO and GR and functionalized hybrid materials with MD10.

Subsequently, the morphology and structural properties of the prepared GO and GR as well as the hybrid materials are studied using the FESEM images. Figure 4 shows the micrographs of GO, GR and their corresponding hybrid polymeric materials. 

In all the cases, the suspended materials are constituted in sheets of several microns; the regular shapes of flakes are visible. GO sheets present shaped like a cluster of agglomerating flakes with regular and sharp edges of several microns. GR sheets also agglomerate but present smaller size than GO. Moreover, the functionalization with polymer seems to provoke the delamination of GR layers into smaller stacks. 

**Figure 5:**
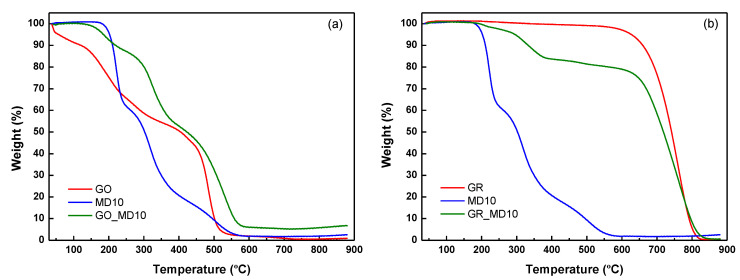
Thermogravimetric (TGA) analysis of (**a**) graphene oxide hybrid sample GO_MD10 compared to GO and MD10 polymers; and (**b**) graphene hybrid sample, GR_MD10 compared to, MD10 and GR.

Next, the attachment of polymers to carbonaceous materials was also analyzed by thermogravimetry under air atmosphere. Figure 5a shows thermal stabilities of the hybrid sample GO_MD10, compared to GO and MD10 polymer. The degradation of the cationic polymer occurs in three stages [41], whereas the GO degrades in two steps, in addition to the loss of adsorbed/absorbed water. The first process at 235 °C is ascribed to the removal of most of functional groups containing oxygen such as labile epoxy, hydroxyl, and carboxylic groups (~36% weight loss) from the GO structure, whereas the second decrease in weight, at around 483 °C, is abrupt and can be associated to the complete thermal decomposition of GO [42]. Table 1 summarizes the decomposition temperatures at 5% weight loss (T_d5_) and the temperatures of the maximum rate of weight loss for each step (T_d_^max1^, T_d_^max2^, T_d_^max3^ and T_d_^max4^) for all the samples. The TGA graph also shows that the GO_MD10 decomposes in several degradation processes. Clearly, the stability of the hybrid sample was in between the stability of GO and the initial polymer, with T_d5_ value of 185 °C. The first degradation process of the GO_MD10 presented a T_d_^max1^= 188 °C, thus at lower temperatures than that of GO and MD10, whereas the rest of the decomposition steps occurred at higher temperatures. This behavior is in agreement with that of other functionalized GO samples, which is associated to the capping of the reactive surface functionalities of GO that are probably sites of decomposition [43,44]. Therefore, the TGA analysis confirms the successful attachment of the cationic polymer onto the GO.

Likewise, the TGA analysis of the graphene-based hybrid sample, GR_MD10, also corroborates the immobilization of the cationic polymer onto the graphene sheets (see Figure 5b and Table 1) of around 10 wt% of polymer. The graphene is stable up to 628 °C with weight loss of 5%. This high stability of graphene results from the strong π-π interactions of the structure. On the other hand, the GR_MD10 started to decompose at 296 °C due to the present of polymer and showed four degradation stages. The first three processes are associated to the polymer immobilized on the graphene, while the last stage at T_d_^max^ = 762 °C corresponds to the complete degradation of graphene sheets. 

**Figure 6:**
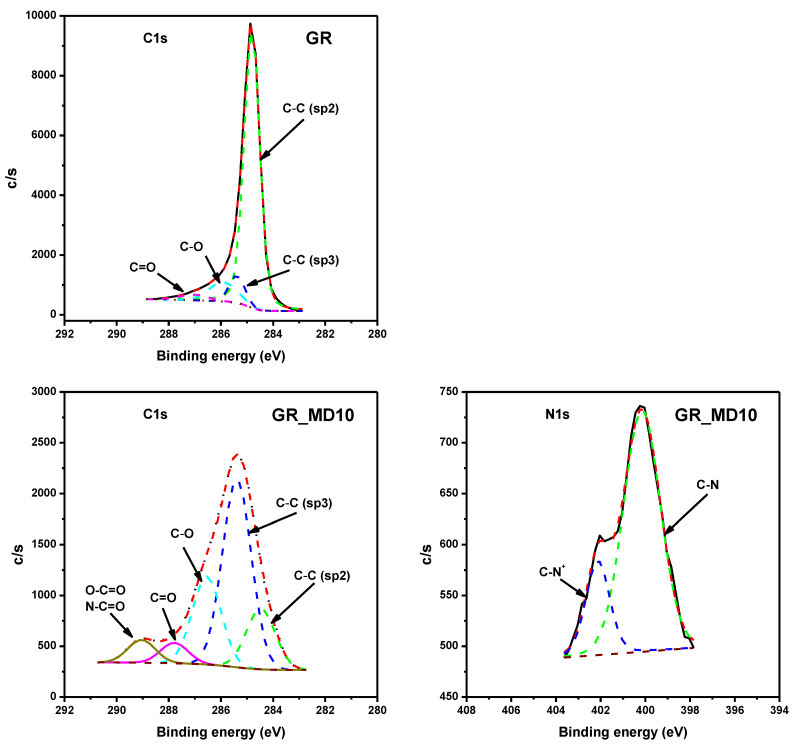
C1s and N1s XPS spectra of GO and GO_MD10.

**Figure 7:**
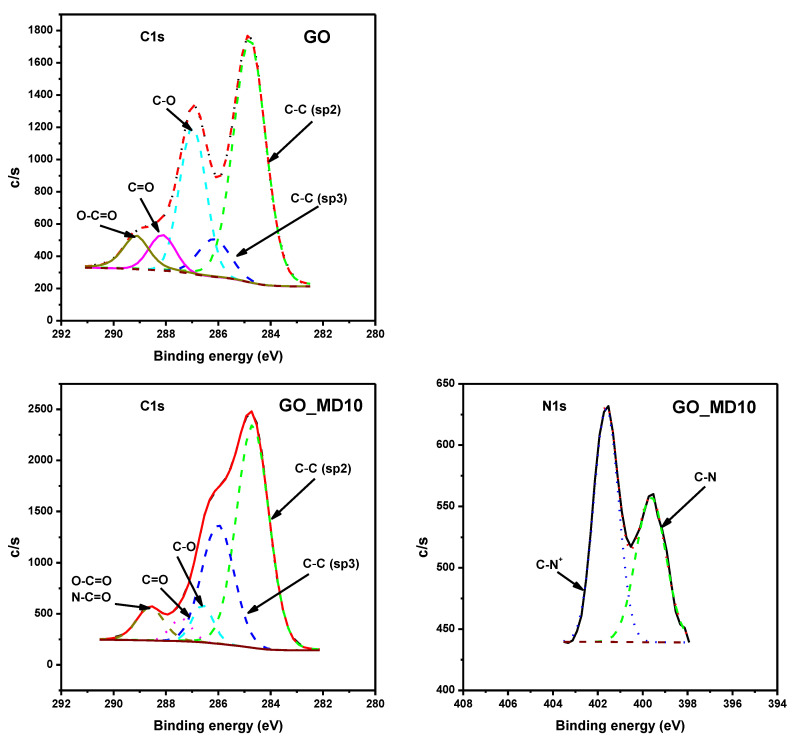
C1s and N1s XPS spectra of GR and GR_MD10.

Moreover, the surface of all materials was analyzed by XPS to confirm the attachment of polymer. Figure 6 and Figure 7 display the C1s profiles of GO and GR and their hybrid materials, respectively. Firstly, C1s profile in GO shows a considerable degree of oxidation with five components that correspond to C atoms of non-oxygenated C=C (sp^2^) of aromatic rings (284.80 eV), the reduced C–C (sp^3^) bonds (286.16 eV), and C atoms in –C–O, C=O and carboxylate, 286.97, 288.14 and 289.14 eV, respectively. In the C1s spectrum of hybrid GO_MD10 system a higher occurrence of sp^3^ C–C as well as a clear decrease on the amount of C–O bond are observed. The N1s spectrum confirmed presence of both O=C–N, and quarternary C–N bond, indicating the attachment of polymers to the carbonaceous surface. 

All these facts, confirm the functionalization of GO and GR with the cationic polymer probably through both, electrostatic interactions and by π-stacking between the hexagonal cells of graphene and graphene oxide and the aromatic ring structure of dopamine [45,46]. 

On the other hand, the C1s XPS spectrum of GR exhibits a dominant presence of the C atoms in the C=C (sp^2^) bonds of the graphenic structure (284.81 eV), with a lower occurrence of C atoms in C–C (sp^3^) bonds at 285.23 eV, which confirms the low impurities observed by Raman measurements. The –C–O and C=O groups are occurring with much lower intensities than in case of GO (285.91 and 287.03 eV, respectively), almost negligible. The C/O ratio is approximately 1.1 for GO while is 60.7 for GR.

The C1s spectrum of GR_MD10 has similar profile than GO_MD10 but with lower proportion of carbonyl groups as expected. Besides, there is also an increment of intensity in the C–C (sp^3^) peak due to the polymer attachment. In the N1s spectrum, GR_DM10 presents O=C–N and C–N^+^ groups, which newly confirm the functionalization of GR. 

The ζ potential in water solutions was also measured to confirm this functionalization. The values are gathered in Table 2 and, as is noticeable, the values of quaternized polymer present high positive charge according to those previously obtained [33]. In contrast, GO and GR present negative values due to their preparation methods and their values are also in agreement to reported values [47,48]. The negative value of GR means that its flakes size is large (>0.46 mm^2^); since only the absolute value of zeta potential, which is more than 30 mV, can ensure its good dispersion stability [49]. 

The functionalization with MD10 makes the carbonaceous materials positively charged and also form stable dispersions. In the case of GO_MD10, this positive charge is lower than for GR_MD10, which could be explained by the stronger negative charge of GO in comparison with GR and the higher proportion of –COO^−^ that could compensate the positive charge of polymer MD10 before the attachment by the catechol groups. 

**Figure 8:**
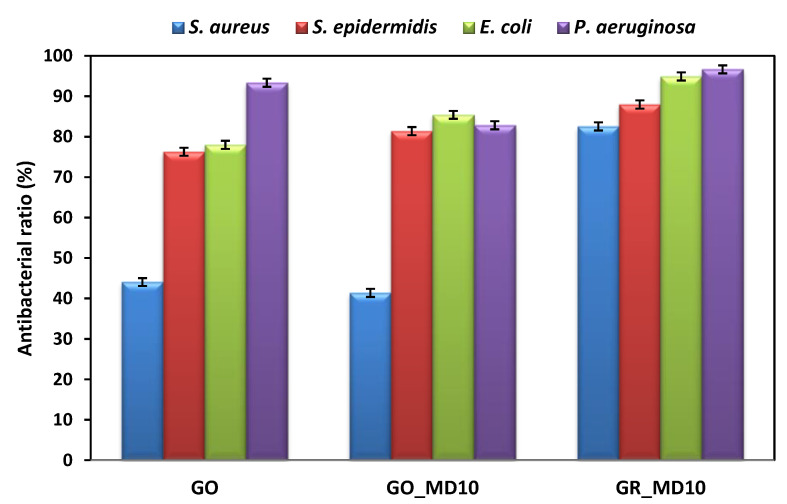
Antibacterial ratio of hybrid materials and GO against bacteria.

As mentioned before, the charges are also important in the antimicrobial response of materials. The antimicrobial behavior of the cationic MD10 polymer was previously evaluated and their minimal inhibitory concentration (MIC) values ranging from 8–64 μg/mL against the tested bacteria [33,50]. Having this in mind, the antimicrobial evaluation of carbon-based materials is performed by contact killing of material (500 µg/mL) and bacteria during 24 h under soft moving. Figure 8 shows the antibacterial ratio against Gram-positive (*S. aureus* and *S. epidermidis*) and Gram-negative (*P. aeruginosa* and *E. coli*) bacteria for GO and GR derivatives including also GO, respectively. It is noticeable that the activities of GO against bacteria is slightly improved with the incorporation of MD10 except against *P. aeruginosa* and *S. aureus* bacteria. Remarkably, hybrid GR_MD10 presents the best results of all, which can be explained by the higher positive charge of the system. It is important to note, that in the case of GR, it was not possible to obtain stable water dispersion during 24 h and then GR sheets did not kill the bacteria at the tested concentration. The incorporation of the cationic polymer significantly improves the water stability, also providing antimicrobial activity. Likewise, the graphene hybrid sample is also more effective against Gram-negative than Gram-positive bacteria. This could be explained by the difference on composition and thickness of the corresponding bacterial cell membranes. 

## 4. Conclusions

The successful functionalization of graphene oxide and graphene with the quaternized statistical copolymer P(MTA90-co-DOMA10), MD10, was confirmed by FTIR, Raman spectroscopy and XPS and the products were further evaluated as antibacterial materials. The structural characterization performed by FTIR confirmed the presence of C=O groups (from MTA) in both hybrid GO_MD10 and GR_MD10 systems. The analysis of the Raman spectra confirmed the functionalization of GO with the cationic polymer through the presence of a new band corresponding to the C–O–C bonds from the ring of DOMA and the graphitic structure of GO, but inducing no changes in the GO structure. In the case of GR, the incorporation of the cationic polymer onto the GR sheets resulted in a structural modification, probably due to the intercalation of the cationic polymer between the graphitic layers and the subsequent formation of graphenic stacks with less number of layers. The functionalization of GR and GO by MD10 quaternized copolymer was also evidenced by the significant change of superficial charge from negative to highly positive values determined by the potential zeta analysis. Moreover, due to the positive charge obtained with the attachment, the hybrid polymeric materials presented good antimicrobial activity against Gram-positive and Gram-negative bacteria, especially in the case of graphene, which activity in water solution is insignificant. 

## Figures and Tables

**Table 1:** Characteristic parameters of the thermal degradation of hybrid materials, the decomposition temperatures at 5% weight loss (T_d5_) and the temperatures of the maximum rate of weight loss for each step (T_d_^max1^, T_d_^max2^, T_d_^max3^ and T_d_^max4^).

Material	T_d5_ (°C)	T_d_^max1^ (°C)	T_d_^max2^ (°C)	T_d_^max3^ (°C)	T_d_^max4^ (°C)
MD10	202	221	316	511	-
GO	158	235	483	-	-
GO_MD10	185	188	320	533	-
GR	628	760	-	-	-
GR_MD10	296	210	347	484	762

**Table 2:** ζ potentials of the different materials.

Material	ζ Potential (mV)
MD10	71 ± 5
GO	−36 ± 3
GO_MD10	35 ± 5
GR	−30 ± 3
GR_MD10	61 ± 4

## References

[1] Shi L., Chen J., Teng L., Wang L., Zhu G., Liu S., Luo Z., Shi X., Wang Y., Ren L. (2016). The antibacterial applications of graphene and its derivatives. Small.

[2] Zhu J., Wang J., Hou J., Zhang Y., Liu J., Van der Bruggen B. (2017). Graphene-based antimicrobial polymeric membranes: A review. J. Mater. Chem. A.

[3] Zou X., Zhang L., Wang Z., Luo Y. (2016). Mechanisms of the antimicrobial activities of graphene materials. J. Am. Chem. Soc..

[4] Zhang Z., Zhang J., Zhang B., Tang J. (2013). Mussel-inspired functionalization of graphene for synthesizing ag-polydopamine-graphenenanosheets as antibacterial materials. Nanoscale.

[5] Tiraferri A., Vecitis C.D., Elimelech M. (2011). Covalent binding of single-walled carbon nanotubes to polyamide membranes for antimicrobial surface properties. ACS Appl. Mater. Interfaces.

[6] Santos C.M., Tria M.C., Vergara R.A., Ahmed F., Advincula R.C., Rodrigues D.F. (2011). Antimicrobial graphene polymer (pvk-go) nanocomposite films. Chem. Commun. (Camb.).

[7] Santos C.M., Milagros Cui K., Ahmed F., Tria M.C.R., Vergara R.A.M.V., de Leon A.C., Advincula R.C., Rodrigues D.F. (2012). Bactericidal and anticorrosion properties in pvk/mwnt nanocomposite coatings on stainless steel. Macromol. Mater. Eng..

[8] Pangilinan K.D., Santos C.M., Estillore N.C., Rodrigues D.F., Advincula R.C. (2013). Temperature-responsiveness and antimicrobial properties of cnt-pnipam hybrid brush films. Macromol. Chem. Phys..

[9] Aslan S., Deneufchatel M., Hashmi S., Li N., Pfefferle L.D., Elimelech M., Pauthe E., Van Tassel P.R. (2012). Carbon nanotube-based antimicrobial biomaterials formed via layer-by-layer assembly with polypeptides. J. Colloid Interface Sci..

[10] Mejias Carpio I.E., Santos C.M., Wei X., Rodrigues D.F. (2012). Toxicity of a polymer-graphene oxide composite against bacterial planktonic cells, biofilms, and mammalian cells. Nanoscale.

[11] Hegab H.M., ElMekawy A., Zou L., Mulcahy D., Saint C.P., Ginic-Markovic M. (2016). The controversial antibacterial activity of graphene-based materials. Carbon.

[12] Placha D., Jampilek J. (2019). Graphenic materials for biomedical applications. Nanomaterials.

[13] Pham V.T.H., Truong V.K., Quinn M.D.J., Notley S.M., Guo Y., Baulin V.A., Al Kobaisi M., Crawford R.J., Ivanova E.P. (2015). Graphene induces formation of pores that kill spherical and rod-shaped bacteria. ACS Nano.

[14] Sawangphruk M., Srimuk P., Chiochan P., Sangsri T., Siwayaprahm P. (2012). Synthesis and antifungal activity of reduced graphene oxide nanosheets. Carbon.

[15] Akhavan O., Ghaderi E., Esfandiar A. (2011). Wrapping bacteria by graphene nanosheets for isolation from environment, reactivation by sonication, and inactivation by near-infrared irradiation. J. Phys. Chem. B.

[16] Di Giulio M., Zappacosta R., Di Lodovico S., Di Campli E., Siani G., Fontana A., Cellini L. (2018). Antimicrobial and antibiofilm efficacy of graphene oxide against chronic wound microorganisms. Antimicrob. Agents Chemother..

[17] Hou W.-C., Lee P.-L., Chou Y.-C., Wang Y.-S. (2017). Antibacterial property of graphene oxide: The role of phototransformation. Environ. Sci. Nano.

[18] Romero M.P., Marangoni V.S., de Faria C.G., Leite I.S., Silva C., Maroneze C.M., Pereira-da-Silva M.A., Bagnato V.S., Inada N.M. (2019). Graphene oxide mediated broad-spectrum antibacterial based on bimodal action of photodynamic and photothermal effects. Front. Microbiol..

[19] Lim H.N., Huang N.M., Loo C.H. (2012). Facile preparation of graphene-based chitosan films: Enhanced thermal, mechanical and antibacterial properties. J. Non Cryst. Solids.

[20] Park S., Ruoff R.S. (2009). Chemical methods for the production of graphenes. Nat. Nanotechnol..

[21] Kumar P., Huo P., Zhang R., Liu B. (2019). Antibacterial properties of graphene-based nanomaterials. Nanomaterials.

[22] Zhang P., Wang H., Zhang X., Xu W., Li Y., Li Q., Wei G., Su Z. (2015). Graphene film doped with silver nanoparticles: Self-assembly formation, structural characterizations, antibacterial ability, and biocompatibility. Biomater. Sci..

[23] Tian T., Shi X., Cheng L., Luo Y., Dong Z., Gong H., Xu L., Zhong Z., Peng R., Liu Z. (2014). Graphene-based nanocomposite as an effective, multifunctional, and recyclable antibacterial agent. ACS Appl. Mater. Interfaces.

[24] Cobos M., De-La-Pinta I., Quindos G., Fernandez M.J., Fernandez M.D. (2020). Graphene oxide-silver nanoparticle nanohybrids: Synthesis, characterization, and antimicrobial properties. Nanomaterials.

[25] Zhao R., Kong W., Sun M., Yang Y., Liu W., Lv M., Song S., Wang L., Song H., Hao R. (2018). Highly stable graphene-based nanocomposite (GO-PEI-Ag) with broad-spectrum, long-term antimicrobial activity and antibiofilm effects. ACS Appl. Mater. Interfaces.

[26] Raja A., Selvakumar K., Rajasekaran P., Arunpandian M., Ashokkumar S., Kaviyarasu K., Asath Bahadur S., Swaminathan M. (2019). Visible active reduced graphene oxide loaded titania for photodecomposition of ciprofloxacin and its antibacterial activity. Colloids Surf. Physicochem. Eng. Asp..

[27] Hsueh Y.H., Hsieh C.T., Chiu S.T., Tsai P.H., Liu C.Y., Ke W.J. (2019). Antibacterial property of composites of reduced graphene oxide with nano-silver and zinc oxide nanoparticles synthesized using a microwave-assisted approach. Int. J. Mol. Sci..

[28] Archana S., Kumar K.Y., Jayanna B.K., Olivera S., Anand A., Prashanth M.K., Muralidhara H.B. (2018). Versatile graphene oxide decorated by star shaped zinc oxide nanocomposites with superior adsorption capacity and antimicrobial activity. J. Sci. Adv. Mater. Devices.

[29] Nguyen H.N., Nadres E.T., Alamani B.G., Rodrigues D.F. (2017). Designing polymeric adhesives for antimicrobial materials: Poly(ethylene imine) polymer, graphene, graphene oxide and molybdenum trioxide - a biomimetic approach. J. Mater. Chem. B.

[30] Mazaheri M., Akhavan O., Simchi A. (2014). Flexible bactericidal graphene oxide–chitosan layers for stem cell proliferation. Appl. Surf. Sci..

[31] Liu T., Liu Y., Liu M., Wang Y., He W., Shi G., Hu X., Zhan R., Luo G., Xing M. (2018). Synthesis of graphene oxide-quaternary ammonium nanocomposite with synergistic antibacterial activity to promote infected wound healing. Burn. Trauma.

[32] Tu Q., Tian C., Ma T., Pang L., Wang J. (2016). Click synthesis of quaternized poly(dimethylaminoethyl methacrylate) functionalized graphene oxide with improved antibacterial and antifouling ability. Colloids Surf. B Biointerfaces.

[33] Chiloeches A., Echeverría C., Cuervo-Rodríguez R., Plachà D., López-Fabal F., Fernández-García M., Muñoz-Bonilla A. (2019). Adhesive antibacterial coatings based on copolymers bearing thiazolium cationic groups and catechol moieties as robust anchors. Prog. Org. Coat..

[34] Hummers W.S., Offeman R.E. (1958). Preparation of graphitic oxide. J. Am. Chem. Soc..

[35] Guadagno L., Raimondo M., Vertuccio L., Mauro M., Guerra G., Lafdi K., De Vivo B., Lamberti P., Spinelli G., Tucci V. (2015). Optimization of graphene-based materials outperforming host epoxy matrices. RSC Adv..

[36] Díez-Betriu X., Álvarez-García S., Botas C., Álvarez P., Sánchez-Marcos J., Prieto C., Menéndez R., de Andrés A. (2013). Raman spectroscopy for the study of reduction mechanisms and optimization of conductivity in graphene oxide thin films. J. Mater. Chem. C.

[37] Kim S.-G., Park O.-K., Lee J.H., Ku B.-C. (2013). Layer-by-layer assembled graphene oxide films and barrier properties of thermally reduced graphene oxide membranes. Carbon Lett..

[38] Vidano R.P., Fischbach D.B., Willis L.J., Loehr T.M. (1981). Observation of raman band shifting with excitation wavelength for carbons and graphites. Solid State Commun..

[39] Hao Y., Wang Y., Wang L., Ni Z., Wang Z., Wang R., Koo C.K., Shen Z., Thong J.T.L. (2010). Probing layer number and stacking order of few-layer graphene by raman spectroscopy. Small.

[40] Malard L.M., Pimenta M.A., Dresselhaus G., Dresselhaus M.S. (2009). Raman spectroscopy in graphene. Phys. Rep..

[41] Chiloeches A., Echeverría C., Fernández-García M., Muñoz-Bonilla A. (2019). Influence of polymer composition and substrate on the performance of bioinspired coatings with antibacterial activity. Coatings.

[42] Gurzęda B., Florczak P., Wiesner M., Kempiński M., Jurga S., Krawczyk P. (2016). Graphene material prepared by thermal reduction of the electrochemically synthesized graphite oxide. RSC Adv..

[43] Thomas H.R., Phillips D.J., Wilson N.R., Gibson M.I., Rourke J.P. (2015). One-step grafting of polymers to graphene oxide. Polym. Chem..

[44] Larciprete R., Fabris S., Sun T., Lacovig P., Baraldi A., Lizzit S. (2011). Dual path mechanism in the thermal reduction of graphene oxide. J. Am. Chem. Soc..

[45] Faure E., Falentin-Daudré C., Jérôme C., Lyskawa J., Fournier D., Woisel P., Detrembleur C. (2013). Catechols as versatile platforms in polymer chemistry. Prog. Polym. Sci..

[46] Kaminska I., Das M.R., Coffinier Y., Niedziolka-Jonsson J., Sobczak J., Woisel P., Lyskawa J., Opallo M., Boukherroub R., Szunerits S. (2012). Reduction and functionalization of graphene oxide sheets using biomimetic dopamine derivatives in one step. ACS Appl. Mater. Interfaces.

[47] Vallés C., Drummond C., Saadaoui H., Furtado C.A., He M., Roubeau O., Ortolani L., Monthioux M., Pénicaud A. (2008). Solutions of negatively charged graphene sheets and ribbons. J. Am. Chem. Soc..

[48] Zhu H., Gao L., Jiang X., Liu R., Wei Y., Wang Y., Zhao Y., Chai Z., Gao X. (2014). Positively charged graphene oxide nanoparticle: Precisely label the plasma membrane of live cell and sensitively monitor extracellular ph in situ. Chem. Commun. (Camb.).

[49] Yi M., Shen Z., Liang S., Liu L., Zhang X., Ma S. (2013). Water can stably disperse liquid-exfoliated graphene. Chem. Commun. (Camb.).

[50] Tejero R., López D., López-Fabal F., Gómez-Garcés J.L., Fernández-García M. (2015). Antimicrobial polymethacrylates based on quaternized 1,3-thiazole and 1,2,3-triazole side-chain groups. Polym. Chem..

